# The Gut Microbiota of Marine Fish

**DOI:** 10.3389/fmicb.2018.00873

**Published:** 2018-05-04

**Authors:** Sian Egerton, Sarah Culloty, Jason Whooley, Catherine Stanton, R. Paul Ross

**Affiliations:** ^1^School of Microbiology, University College Cork, Cork, Ireland; ^2^School of Biological, Earth and Environmental Sciences, University College Cork, Cork, Ireland; ^3^Environmental Research Institute, University College Cork, Cork, Ireland; ^4^Bio-marine Ingredients Ireland Ltd., Killybegs, Ireland; ^5^Teagasc Research Centre, Fermoy, Ireland; ^6^APC Microbiome Ireland, Teagasc and University College Cork, Cork, Ireland

**Keywords:** intestinal bacteria, microbial ecology, metagenomics, dietary intervention, salmon, trophic levels, probiotics and prebiotics, aquaculture

## Abstract

The body of work relating to the gut microbiota of fish is dwarfed by that on humans and mammals. However, it is a field that has had historical interest and has grown significantly along with the expansion of the aquaculture industry and developments in microbiome research. Research is now moving quickly in this field. Much recent focus has been on nutritional manipulation and modification of the gut microbiota to meet the needs of fish farming, while trying to maintain host health and welfare. However, the diversity amongst fish means that baseline data from wild fish and a clear understanding of the role that specific gut microbiota play is still lacking. We review here the factors shaping marine fish gut microbiota and highlight gaps in the research.

## A Historical Overview

Fish and other marine animals have a unique and intimate interaction with their surrounding environment and, in turn, with the microorganisms that co-exist there. The world’s oceans are teeming with microorganisms. It is estimated that 3.6 × 10^30^ microbial cells account for more than 90% of the total oceanic biomass, while the number of viral particles may be one hundred fold greater (International Council for the Exploration of the Sea [ICES], 2011). The relationship that fish have with surrounding microorganisms can be mutualistic or pathogenic. Like humans and other mammals, fishes’ associated symbiotic gut microbiota play a role in nutritional provisioning, metabolic homeostasis and immune defence (Gómez and Balcázar, 2008; Sullam et al., 2012).

Fish originated over 600 million years ago and include nearly half of all extant vertebrates (Nelson, 2006; Sullam et al., 2012). Over three billion people around the world depend on fish for at least 20% of their protein intake and approximately 20 kg of fish is consumed per capita per annum (Food and Agriculture Organisation of the United Nations [FAO], 2016). Wild-caught fisheries can no longer support the world’s seafood consumption thus, unsurprisingly, aquaculture is reported to have contributed 43.1% of global fish production in 2013 (Food and Agriculture Organisation of the United Nations [FAO], 2015). The vast diversity that fish contribute to the sub-phylum chordata, our reliance on fish as a food source and the environmental changes that are being inflicted on them highlight the need to consider them in the growing field of host microbial research.

Research into the gut microbiota of fish dates back to the early half of the 20th century but more recently interest in this area has grown at a significant rate coinciding with the expansion of the aquaculture industry. Indeed, the first works on this topic were published in the late 1920’s and 1930’s (Reed and Spence, 1929; Gibbons, 1933) and investigated the intestinal and “slime flora” of fish. There were some further exploratory studies during the 1950’s and 60’s; Margolis (1953) investigated the effect of fasting on the intestinal flora, Colwell (1962) examined the intestinal flora of Puget Sound fish and Simidu and Hasuo (1968) examined the salt dependency of fish flora. In the following decade, the studies became more applied, with interest in how the gut microbiota changed with diet (Sera and Ishida, 1972a), how the microbiota changed in farmed fish (Gilmour et al., 1976) and how animals succumbed to infection (Boulanger et al., 1977; Olivier et al., 1981).

In the early 1990’s the first reviews on this topic were published (Cahill, 1990; Ringø et al., 1995). They provided a comprehensive overview of the studies to date; however, they consequentially reported that bacterial levels in the gut of fish were low and appeared to be derived from the surrounding environment or diet (Cahill, 1990; Ringø et al., 1995). These conclusions were made based on research using culture-dependent methods but we now know that no more than 10% of microorganisms could be isolated and cultured under such laboratory conditions as were used then (Amann et al., 1995). Analytical techniques have evolved significantly since then and it is now reported that cultivable microorganisms represent < 0.1% of the total microbial community in the gastrointestinal (GI) tract of some species of fish (Zhou et al., 2014). Despite this, many recent studies continue to report results obtained through culture-based approaches, inferring microbiota function from data derived from bacterial growth studies performed under artificial environmental conditions (Clements et al., 2014). Today a wide variety of culture-independent techniques are available for analysing fish microbiota. These have been discussed in detail in some recent reviews (Zhou et al., 2014; Tarnecki et al., 2017) Briefly, they include quantitative real-time PCR (qPCR), used for quantitative analysis of taxa; clone libraries for identification of microbiota composition; finger-printing methods such as temporal temperature gradient electrophoresis (TTGE) and denaturing gradient gel electrophoresis (DGGE), and fluorescent *in situ* hybridization (FISH) used to determine the abundance of particular taxa, total microbial levels and assess bacterial–host interactions at the mucosal brush border (Zhou et al., 2014; Wang et al., 2017). Next-generation sequencing is the latest method of molecular analysis. It is beginning to be used more frequently in studies on fish and Ghanbari et al. (2015) have discussed its potential in this field, including the opportunity for rapid and cost-effective acquisition of in-depth and accurate sequence data that provide greater information on even low abundance microbiota as well as the genetic and metabolic potential of the species present.

With the development of these new molecular techniques and the exponential growth of aquaculture, the research of fish gut microbiota has expanded dramatically over the previous decades. In this review, we focus on the gut microbiota of marine species. We have included anadromous salmonids in our discussions but do not focus on them or the novel changes that these fish experience in their gut microbiota as they develop and move across habitats. This is an area which has thus far been poorly understood but is receiving new interest in some recently published articles; Llewellyn et al. (2016), Dehler et al. (2017), and Rudi et al. (2018). Even when looking specifically at saltwater fish, the diversity is enormous. In this review, we discuss the trends and supporting findings in the current literature, but also highlight the contradictory studies that are inevitable within such a diverse group. Overall, the purpose of this review is to provide an overview of the fish alimentary canal, the gut microbiota within it and how the diversity of these communities develops with life stage and is affected by factors including trophic level, season and captive-state. Finally, we review the latest research that investigates the dietary manipulation of gut microbiota in aquaculture species and discuss future perspectives.

## The Fish Alimentary Canal

**FIGURE 1:**
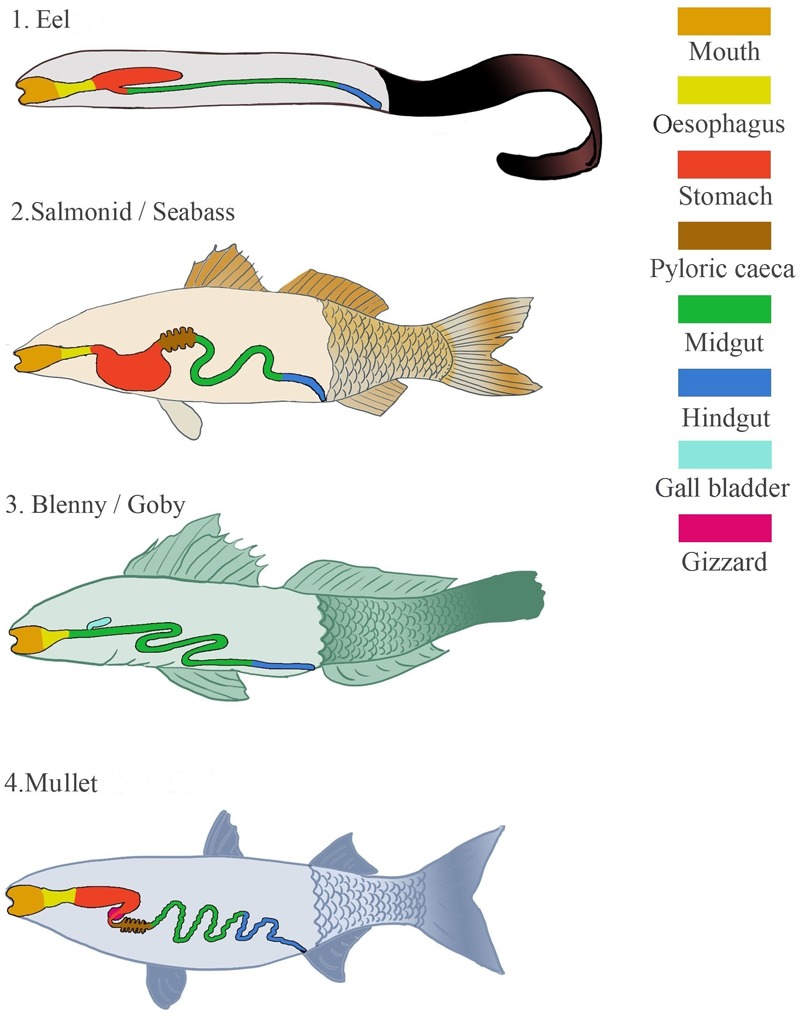
Diagrammatic representation of the different types of digestive systems that can be found in marine fish, including digestive organs that may or may not be present.

There is no single blue print for the alimentary canal of a fish; fish biology varies greatly with differing life histories, ecology and environmental factors. Philtre feeders, parasites and predators as well as herbivorous and carnivorous fish exist and each has an appropriately adapted digestive system. Regardless of diet, the gut of some fish consists simply of a short tubular intestine, e.g., parrotfish, *Scarus radicans* (Horn et al., 2006). However, the majority of fish alimentary canals are divided into topographical regions with unique roles. All fish alimentary canals begin with the buccal and pharyngeal cavities of the head-gut. From here, the gut can be loosely divided into the fore-, mid- and hind-gut which include various digestive organs that particular fish either possess or lack. The foregut, beginning at the posterior edge of the gills, often consists of the oesophagus, stomach and pylorus. However, it is estimated that 20% of fish species lack a true stomach (Wilson and Castro, 2010). Species that have evolved such simple digestive tracts include fish in the Gobiidae and Blennidae families (**Figure 1**). This lack of stomach in some species may be counteracted by other adaptations such as well-developed pharyngeal teeth, pharyngeal pockets, secretory glands in the oesophagus or a muscular gizzard (James, 1988; Kapoor and Khawna, 1993; Stevens and Hume, 2004). When the stomach is present it is usually one of three shapes; straight, U-shaped, or Y-shaped with a gastric cecum (**Figure 1**). Straight stomachs are relatively rare but can be found in some freshwater species as well as marine fish such as mullet, *Mugil*, anchovy, *Engraulis*, and menhaden, *Brevoortia*. The U-shaped stomach is more frequently seen and is common in omnivores and carnivores such as seabass, *Dicentrarchus*, and salmonids. The Y-shaped stomach is proposed to be an adaptation of macrophagous predatory fish for storage of large pieces of food and is found in eels, *Anguilla* (Stevens and Hume, 2004).

Generally no definitive distinction exists between the mid- and hind-gut. However, the former is the longest portion of the gut, which includes the pyloric ceca when present. The mid-gut is where the majority of digestion occurs and the pyloric ceca are thought to be organs acquired to produce a greater surface area for absorption. Although not always obvious, this section often ends with an increase in tube diameter, indicating the beginning of the hindgut (distal intestine and anus). Fish intestines vary dramatically in length. When longer than the visceral cavity, the intestines are coiled in a loop unique to each species. Gut length is loosely associated with diet and as a guide is three times longer than body length in herbivorous fish, one to three times in omnivores and approximately equal in carnivores (Bone et al., 1995; Karachle and Stergiou, 2010).

## Development of the Gut Microbiota in Fish

Microbial colonisation of fish larvae originates from the eggs, the surrounding water and the first feed. Some initial studies investigating bacteria associated with fish eggs suggested that the dominating species at this point included *Cytophaga, Flavobacterium*, and *Pseudomonas* (Bell et al., 1971; Yoshimizu et al., 1980; Austin, 1982). While some recent studies provide correlating results (Kubilay et al., 2009), others differ completely (Romero and Navarrete, 2006; McIntosh et al., 2008). Even some early studies recognised that inter-species variation existed. For example, Hansen and Olafsen (1989) observed differences in the bacterial colonisation of cod, *Gadus morhua L.*, and halibut, *Hippoglossus hippoglossus*, eggs. The initial colonising bacteria are now accepted as species-specific, with differences controlled by variation in binding glycoproteins on the egg surface (Larsen, 2014). In addition, the microbiota of the surrounding water dictates what bacteria encounter the eggs and consequently have the opportunity to colonise. Upon hatching, sterile larvae take in the chorion-associated bacteria, which become the first colonisers of the developing gastrointestinal tract (GIT). Subsequent inhabiting bacteria are acquired when the fish larvae begin to drink water to control osmoregulation and the microbiota then becomes further diversified through feeding (Hansen and Olafsen, 1999). To begin, the GIT of newly hatched larvae tend to contain few bacteria (Ringø et al., 1991). Numerous studies have shown that diet is influential in shaping the gut microbial community and from first feeding substantial diversification occurs (Blanch et al., 1997; Korsnes et al., 2006; Reid et al., 2009; Lauzon et al., 2010). Interestingly, like in humans (Yatsunenko et al., 2012), it appears the diversity of bacteria increases as fish develop. In Ringø and Birkbeck’s (1999) review of the *Intestinal microflora of fish larvae and fry*, they summarised 24 studies that reported the bacterial genera in the intestinal tract of freshwater and marine fish at the larval and fry stages. In the 11 marine species, the bacteria most frequently reported were *Vibrio* (15 times), *Pseudomonas* (9), *Cytophaga* (8), *Flavobacterium* (7) and the family Enterobacteriaceae (7). On average, the studies reported three to four genera/families (**Table 1**). A comparison of the gut microbiota of 12 (adult) bony fish found bacteria representing 17 phyla, with most species having between 7 and 15 phyla, a far higher average than in the review of egg and larvae microbiota. While the microbial community changes with life stage and habitat, a relatively stable gut microbiota is established within the first 50 days of life for many species (McIntosh et al., 2008; Larsen, 2014). A decisive study with zebrafish, *Danio rerio*, demonstrated this, reporting that a core microbial community is supported through host system selective pressures regardless of environmental parameters (Roeselers et al., 2011).

**Table 1:** Bacterial species isolated from the intestinal tracts of marine fish species at larval and fry life stages.

Fish species	Bacterial genera	Reference
Atlantic cod, *Gadus morhua*	*Vibrio/Aeromonas* *Aeromonas, Pseudomonas, Cytophaga/Flexibacter, Lactobacillus*	Strøm and Olafsen, 1990 Strøm and Ringø, 1993
Atlantic halibut, *Hippoglossus hippoglossus*	*Cylophaga/Flexibacter/Flavobacterium*, *Vibrio/Aeromonas Vibrio/Aeromonas*	Bergh et al., 1994 Bergh, 1995
Dover sole, *Solea solea*	*Pseudomonas/Alcaligenes, Vibrio/anaergenic Aeromonas, Moraxella, Enterobacteriaceae, Flavobacterium/Cytophaga, Moraxella, coryneforms*	Campbell and Buswell, 1983
Turbot, *Scophthalmus maximus*	*Vibrionaceae*	Nicolas et al., 1989
	*Vibrio alginolyticus, Aeromonas* *Vibrio pelagius* *Vibrio alginolyticus, V. natrigenes, V. anguillarum, V. fluvilis, V. pelagius, Aeromonas caviae, Acinetobacter*	Gatesoupe, 1990 Blanch et al., 1997 Munro et al., 1993
	*Vibrio alginolyticus, V. anguillarum, V. campelii, V. fluvialis, V. furnissi, V. harveyii, V. natrigenes, V. nereis, V. ordali, V. pelagius, V. splendidus, Vibrio, Aeromonas, Pseudomonas/Alcaligenes, Flavobacterium/Cytophaga, Enterobacteriaceae, Acinetobacter, Photobacterium, Moraxella*	Munro et al., 1994
	*Aeromonas, Vibrio, Enterobacteriaceae, Cytophaga, Micrococcus, Staphylococcus, coryneforms*	Ringø et al., 1996
	*Oxidative Gram-negative rods, V. natriegens, V. eplagius, V. sophalmis, V. splendidus, V. mediterranei, V. anguillarum, V. alginolyticus*	Blanch et al., 1997
	*Acinetobacter, Moraxella, Vibrio*	Gatesoupe et al., 1997
Herring, *Clupea harengus*	*Pseudomonas/Alteromonas, Flavobacterium*	Hansen et al., 1992
Rockfish, *Sebastes schlegeli*	*Vibrio, V. anguillarum, V. alginolyticus, Pseudomonas, Acinetobacter, Flavobacterium/Cytophaga*	Tanasomwang and Muroga, 1989
Red seabream, *Pagrus major*	*Aeromonas, Vibrio, Pseudomonas, Enterobacteriaceae, Cytophaga*	Muroga et al., 1987
Black seabream, *Acanthopagrus schlegeli*	*Aeromonas, Vibrio, Pseudomonas, Enterobacteriaceae, Cytophaga*	Muroga et al., 1987
Milkfish, *Chanos chanos*	*Pseudomonas, Vibrio, Enterobacteriaceae*	Fernandez et al., 1996
Seabass, *Dicentrarchus labrax*	*Vibrio, Acinetobacter, Moraxella, Enterobacteriaceae*	Gatesoupe et al., 1997
Wolffish, *Anarhichas lupus*	*Carnobacterium divergens*	Ringø and Johnsen, unpublished data

*Taken from Ringø and Birkbeck, 1999.*

## Structure of the Fish Gut Microbiota

The fish microbiome can be diverse, including protoctista, fungi, yeasts, viruses, and members of the Bacteria and Archaea (Merrifield and Rodiles, 2015). Bacteria are the dominant microbiota of the fish intestine (Rombout et al., 2011) however, and have been almost the sole focus of research in this field thus far. Recent research has shown that fish hindgut microbial communities closely resemble those of mammals much more so than their surrounding environmental microbial communities (Ktari et al., 2012). Despite this, in mammals the dominant gut microbiota are anaerobes from the phyla Bacteroidetes and Firmicutes (Lozupone et al., 2012) whereas Proteobacteria are the prominent microbial phyla found in the fish GIT (Rombout et al., 2011). Proteobacteria, in addition to Bacteroidetes and Firmicutes, comprise 90% of the fish intestinal microbiota of the different species studied thus far (Ghanbari et al., 2015).

The density, composition and function of the microbiota change in the different sections of the fish GIT (Clements et al., 2014). Furthermore, there is a distinction between the allochthonous and autochthonous communities (Nayak, 2010; Banerjee and Ray, 2017). Allochthonous are the free-living, transient microbiota associated with the digesta, whereas, autochthonous microbiota colonise the mucosal surface of the digestive tract and make up the core community. The density of viable aerobic and anaerobic bacteria usually range from 10^4^–10^9^ colony forming units (CFU) g^-1^ of intestinal content, respectively (Skrodenytė-ArbaČIauskiene, 2007). This is notably lower than that of warm-blooded animals which are generally orders of magnitude higher (Nayak, 2010). Similar to higher vertebrates, the densest population of microbes in teleost fish is located in the GIT. Previous studies have found increasing population sizes running distally along the GIT. Aerobic heterotrophs in the GIT of yellowtail (*Seriola* sp.) increased from 2 × 10^4^ bacteria g^-1^ in the pyloric caeca and 2.5 × 10^5^ bacteria g^-1^ in the stomach, finally, to 6.5 × 10^4^ to 5.9 × 10^6^ bacteria g^-1^ in the intestine (Sakata et al., 1978). This trend was also observed in herring, *Clupea harengus*, larvae (Hansen et al., 1992) and juvenile Dover sole, *Solea solea*, though not adults (MacDonald et al., 1986). The results of an analysis of the occurrence and distribution of enzyme-producing bacteria in the proximal, middle, and distal segments of the GIT of four brackish water teleosts (*Scatophagus argus, Terapon jarbua, Mystus gulio*, and *Etroplus suratensis*) showed that the density generally increased along the GIT (Das et al., 2014). Other studies also found similar trends (Fidopiastis et al., 2006; Ringø et al., 2006; Bakke-McKellep et al., 2007; Hovda et al., 2007; de Paula Silva et al., 2011). Zhou et al. (2007) used 16S rDNA PCR-DGGE fingerprinting to study the autochthonous bacteria of *Lutjanus sebae* and *Ephippus sebae*. In this study, they found that the average number of different bacteria detected in each section increased along the digestive tract. In contrast, the opposite was found by Zhou et al. (2009a) in yellow grouper, *Mycteroperca venenosa*, and no obvious trend was observed in juvenile Atlantic salmon, *Salmo salar* (Navarrete et al., 2009). Numerous factors may have caused the deviating results of these studies; diet, which may be a significant one, will be discussed later in this review.

The community composition between sections of the fish GIT can also vary (Llewellyn et al., 2014). It has been suggested that the autochthonous microbiota can differ in particular, considering the variation in physiological environments between the different parts of the digestive tract (Clements et al., 2014). The stomach is often omitted from gut microbial composition analyses. However, a number of studies have included it in the past using culture-dependent techniques (Sera and Ishida, 1972a,b; Austin and Al-Zahrani, 1988; Ringø, 1993; Ringø et al., 1998; Zhou et al., 2008, 2009b). There are also some more recent studies using culture-independent techniques to compare the microbial community in different gut segments, including the stomach. The dominant phyla in the stomach of gilthead seabream, *Sparus aurata*, were reported as Firmicutes, Proteobacteria and Bacteroidetes (de Paula Silva et al., 2011). However, a later study reported the dominant phyla to be Firmicutes, Proteobacteria, and Actinobacteria (Estruch et al., 2015). Both studies found Vibrionaceae to be a dominant family, reporting the genus *Photobacterium*. Aside from this, Estruch et al. (2015) also reported the family Enterobacteriaceae, the genera *Streptococcus* and *Clostridium* of Firmicutes and the genus *Corynebacterium* of Actinobacteria, whereas de Paula Silva et al. (2011) found bacteria relating to the genus *Vibrio* along with species from the family *Bacillales* of Firmicutes and the genus *Flavobacteriaceae* of Bacteroidetes. Results on stomach microbiota should be treated with caution. These two studies used stomach contents for analysis which is likely to be influenced by transient food. Another study that included analysis of the adherent stomach microbiota found greater diversity of bacteria in the stomach of yellow grouper compared to other sections of the gut (Zhou et al., 2009a). The genera *Proteobacterium, Pantoea*, and *Clostridium* were found in all sections of the yellow grouper gut, whereas the less commonly reported phyla *Deinococcus-Thermus* and *Planctomycete* were found only in the stomach along with uncultured *Streptococcus* sp. and *Enterobacter amnigenus*. Interestingly, not all studies have found significant differences between sections. Although included in analysis, no significant differences in adherent community composition in the stomach and intestine were reported for red emperor snapper, *Lutjanus sebae* (Zhou et al., 2009b).

When a dietary intervention trial was undertaken on Atlantic cod, *Gadus morhua*, differences in gut microbiota were seen between the different diets, but interestingly, within each diet there was variation in dominant species found in the fore- and midgut, and the hindgut (Ringø et al., 2006). Indeed, in fish fed a fishmeal diet, *Psychrobacter* and *Brochothrix* were dominant in the fore- and midgut, while Carnobacteriaceae was dominant in the hindgut microbiota. Interestingly, fish fed the soybean meal and the bioprocessed soybean meal diets had *Psychrobacter* dominating throughout the gut. Variation in dominant species in the fore- mid- and hind-gut were also observed in farmed Atlantic salmon (Hovda et al., 2007). The fore-gut was dominated by Proteobacteria in the genera *Janthinobacterium, Pseudomonas, Acinetobacter*, and *Vibrio*; the mid-gut by the Proteobacteria *Photobacterium phosphoreum* and the genus *Pseudomonas*; while in the hind-gut it was *Vibrio* and *P. phosphoreum* which were present in higher numbers. The differences presented from analyses of different gut segments and gut contents or gut mucus highlights the importance for all studies to report the details of their sample preparation.

Studies investigating the gut microbiota of fish are varied at many levels, including species studied and methods of sample collection and analysis. This can create difficulties when comparing results and extrapolating the true level of diversity. Despite these limitations, results from a comparison which non-uniformly spans a diversity of fish species from over 30 studies revealed the following genera to be the most frequently reported as dominant: *Vibrio* (11 times), *Photobacterium* (10) and *Clostridium* (5) (**Table 2**). In support of these results, a meta-analysis of the gut communities of marine fish revealed that Vibrionales bacteria (which includes the genera *Vibrio* and *Photobacterium*) accounted for 70% of sequence reads (Sullam et al., 2012).

**Table 2:** Dominant bacterial species isolated from the intestinal tracts of marine fish species at different trophic levels.

Trophic level	Fish species	Dominant bacteria genera	Reference
***Herbivores***			
	Butterfish, *Odax pullus*	*Clostridium*	Clements et al., 2007
	Marblefish, *Aplodactylus arctidens*	*Clostridium, Eubacterium desmolans, Papillibacter cinnaminovorans*	Clements et al., 2007
	Parrotfish, *Chlorurus sordidus*	*Vibrio, Photobacterium*	Smriga et al., 2010
	Silver drummer, *Kyphosus sydneyanus*	*Clostridium*	Moran et al., 2005
	Surgeonfish, *Acanthurus nigricans*	Bacteroidetes, non-vibrio Proteobacteria, Firmicutes	Smriga et al., 2010
	Surgeonfish, *Acanthurus sp.*	*Epulopiscium*	Miyake et al., 2015
	Zebraperch, *Hermosilla azurea*	Enterovibrio, Bacteroides, Faecalibacterium, Desulfovibrio	Fidopiastis et al., 2006
***Omnivores***			
	Pinfish, *Lagodon rhomboides*	*Clostridium, Mycoplasma*	Ransom, 2008
		*Photobacterium, Propionibacterium, Staphylococcus, Pseudomonas, Corynebacterium*	Givens et al., 2015
	Long-jawed mudsucker, *Gillichthys mirabilis*	*Mycoplasma*	Bano et al., 2007
***Carnivores***			
	Atlantic cod, *Gadus morhua*	*Clostridium perfringens*	Aschfalk and Müller, 2002
		*Vibrio*	Star et al., 2013
	Atlantic halibut, *Hippoglossus hippoglossus*	Vibrionaceae (larvae, juveniles), *Photobacterium phosphoreum* (adults)	Verner-Jeffreys et al., 2003
	Atlantic salmon, *Salmo salar*	*Acinetobacter junii, Mycoplasma*	Holben et al., 2002
		*Lactobacillus, P. phosphoreum, Lactococcus, Bacillus*	Hovda et al., 2007
	Blackfin icefish, *Chaenocephalus aceratus*	*Photobacterium*	Ward et al., 2009
	Black rockcod, *Notothenia coriiceps*	*Photobacterium, Vibrio*	Ward et al., 2009
	Bluefish, Pomatomus saltatrix	Vibrio, Pseudomonas, Enterobacteraceae	Newman et al., 1972
	Gilthead sea bream, Sparus aurata	Pseudomonas	Floris et al., 2013
	Grass puffer, Fugu niphobles	Vibrio, Pseudomonas, Flavobacterium	Sugita et al., 1989
	Grouper, Epinephelus coioides	Bacillus, Vibrio, Delftia, Psychroacter, Acinetobacter, Pseudomonas	Sun et al., 2009
	Red drum, Sciaenops ocellatus	Mycoplasmataceae	Ransom, 2008
		Photobacterium, Cetobacterium, Clostridiaceae, Vibrio	Givens et al., 2015
	Sea trout, Salmo trutta trutta	Aeromonas sobria, Pseudomonas	Skrodenytė-ArbaČIauskiene et al., 2008
	Siberian sturgeon, Acipenser baerii	Cetobacterium somerae	Geraylou et al., 2013
	Snapper, Lutjanusn bohar	Vibrio, Photobacterium	Smriga et al., 2010
	Southern flounder, Paralichthys lethostigma	Clostridium	Ramirez and Dixon, 2003
		Clostridium	Ransom, 2008
		Photobacterium, Clostridiaceae, Clostridium	Givens et al., 2015
	Speckled trout, Cynoscion nebulosus	Escherichia coli	Ransom, 2008
	Striped bass, Morone saxatilis	Aeromonas, Pseudomonas, Vibrio	MacFarlane et al., 1986
***Zooplanktivores***			
	Cardinalfish, Apogonidae	Vibrionaceae and Pasteurellaceae, Vibrio harveyi, Shewanella sp., Endozoicomonas sp.	Parris et al., 2016
	Damselfish, Pomacentridae	Vibrionaceae and Pasteurellaceae, Vibrio harveyi, Shewanella sp., Endozoicomonas sp.	Parris et al., 2016
	Herring, Clupea harengis	Pseudomonas, Alteromonas	Hansen et al., 1992
		Pseudomonas, Psychrobacter	Curson et al., 2010
	Pipefish, Syngnathus scovelli	Proteobacteria	Ransom, 2008
	Sardines, Sardinella longiceps	Achromobacter, Vibrio, Pseudomonas	Karthiayani and Mahadeva Iyer, 1967
	Atlantic mackerel, Scomber scombrus	Psychrobacter, Vibrio, Shewanella	Svanevik and Lunestad, 2011

*Vibrio*, a diverse genus of the phylum Proteobacteria, is one of the most important bacterial genera in aquaculture, with both pathogenic and probiotic (health-promoting) species (Vandenberghe et al., 2003). *V. anguillarum, V. salmonicida*, and *V. vulnificus* are among the main bacterial pathogens of marine fish and invertebrate species (Austin and Austin, 1999). Pathogenic *Vibrios* commonly infect larvae and can cause sudden and significant mortalities. However, it has been hypothesised that many *Vibrio* species are not true pathogens, but in fact opportunistic pathogens whose virulence is accentuated under intensive aquaculture conditions (Thompson et al., 2004). *Vibrio alginolyticus*, although sometimes pathogenic (Samad et al., 2014; Chen et al., 2015), has been shown *in vivo* to work well as a probiotic for Atlantic salmon, protecting against *Aeromonas salmonicida, Vibrio anguillarum* and *Vibrio ordalii* (Austin et al., 1995). An *in vitro* study found that *Vibri*o sp. Strain NM 10 had an inhibitory effect against the fish pathogen *Pasteurella piscicida* (Sugita et al., 1997). Many *Vibrio* species produce hydrolytic enzymes and in this way they can act as symbionts assisting in the breakdown of dietary components. Strains have been found to produce amylase (Hamid et al., 1979; Gatesoupe et al., 1997), lipase (Gatesoupe et al., 1997; Henderson and Millar, 1998), cellulose (Itoi et al., 2006; Sugita and Ito, 2006) and chitinase (MacDonald et al., 1986) among others (Ray et al., 2012).

*Photobacterium* is also a genus of the phylum Proteobacteria and family Vibrionaceae. This luminous bacteria is commonly found on the surface of healthy fish and was originally associated with light-emitting organs, e.g., *Photobacterium angustum, P. leiognathi* and *P. phosphoreum* (Cahill, 1990). Initially, it was recognised that surface tubules release these bacteria into the digestive tract of the host (Haygood and Distel, 1993). However, since then numerous strains of *Photobacterium* have been found in the GIT of fish species lacking bioluminescent organs (Makemson and Hermosa, 1998; Ward et al., 2009; Smriga et al., 2010). There are also non-luminescent members of the *Photobacterium* genus, such as *P. iliopiscarium* which has been isolated from the intestines of several species of cold-water fish (Onarheim et al., 1994; Urakawa et al., 1999). Many *Photobacterium* act as mutualistic bacteria in the host gut aiding with chitin digestion (MacDonald et al., 1986; Ramesh and Venugopalan, 1989; Itoi et al., 2006). However, some also produce harmful enzymes such as neuraminidases (Sugita et al., 2000). *Photobacterium damselae* is a neuraminidases producer and is a common pathogen for wild and captive fish (Romalde, 2002). There are two sub-species of *P. damselae*; *P. damselae* ssp. *damselae* and *P. damselae* ssp. *piscicida*. The former is associated with skin ulcers, while the latter is the infectious agent of pasteurellosis in fish (Urbanczyk et al., 2011).

*Clostridium* is a very common genus within the phylum Firmicutes. It is a Gram-positive obligatory anaerobe with many pathogenic species. *Clostridium difficile* is a commonly known species of this genus as it is associated with diarrheal disease in humans and animals (Metcalf et al., 2011). However, it has not been widely associated with marine fish, though studies investigating *C. difficile* in fish are limited. One study that did investigate its presence found no *C. difficile* in 107 assorted marine and freshwater fish gut contents (Al Saif and Brazier, 1996). It has previously been isolated from freshwater African cichlids, *Nimbochromis venustus*, with the condition known as “Malawi bloat,” suggesting that if present it is pathogenic in fish (Dixon et al., 1997).

*Clostridium botulinum* is a pathogenic species more frequently associated with marine fish. There are six different type strains (A-F). Fish are susceptible to type E and occasionally B (Mazuet et al., 2016; Uzal et al., 2016). When 117 intestinal samples from rainbow trout, *Oncorhynchus mykiss*, were analysed for *C. botulinum* type E in Finland, 15% were found positive (Hyytiä et al., 1998). Similarly, in a study performed in northern France, the prevalence of *C. botulinum* in marine fish was recorded at 16.6% (Fach et al., 2002). Huss and Pedersen (1979) reported that *C. botulinum* was more common for demersal rather than pelagic marine fish and suggested this was as a result of greater interaction with the sediment. It has been noted that fish are not always affected by *C. botulinum* and can be healthy carriers of the spores (Uzal et al., 2016).

*Clostridium* species often work as mutualistic symbionts with marine hosts, especially herbivorous fish (Clements et al., 2007, 2009). They have been shown to contribute to the host’s nutrition, especially by supplying fatty acids and vitamins (Balcázar et al., 2006). In southern flounder, *Paralichthys lethostigma, Clostridium* along with other Gram-negative genera displayed enzyme activities of acid and alkaline phosphatases, C4 and C8 esterases, C14 lipases, arylamidases and glycosidases (Ramirez and Dixon, 2003). Beyond this natural symbiosis, some species of *Clostridium*, such as *C. butyricum* have been used successfully as a probiotic in aquaculture, enhancing resistance of rainbow trout to vibriosis (Sakai et al., 1995) and stimulating the immune response and improving survival in Japanese flounder, *Paralichthys olivaceus*, (Taoka et al., 2006).

## Diversity of Fish Gut Microbiota

Studies on the gut microbiota of fish have found substantial intra- and inter-species diversity. Factors which influence this diversity include life stage (Hansen and Olafsen, 1999), trophic level (Clements et al., 2007), diet (Cordero et al., 2015), season (Hovda et al., 2012), habitat (Bano et al., 2007), captive-state (Dhanasiri et al., 2011), sex (Dhanasiri et al., 2011), and phylogeny (Miyake et al., 2015). A recent meta-analysis investigating the factors affecting the gut microbiota composition of fish reported that trophic level, habitat and possibly host phylogeny are the most likely influencers (Sullam et al., 2012). In the following sections, we review the literature thus far relating to the effects of trophic level, season and captive state on the gut microbiota of fish.

### Trophic Level

The influence of diet on gut microbiota is a logical link and has been reported numerous times for an array of species (Claesson et al., 2012; Serino et al., 2012; Miyake et al., 2015; Li et al., 2017). Trophic position relates natural diet with evolutionary development and marine fish fill many of these levels. In terms of investigating the relationship of gut microbial composition with trophic level, early studies included flatfish (Liston, 1956) and salmon (Yoshimizu and Kimura, 1976), however, there was also significant interest in herbivores.

The seminal studies of Fishelson et al. (1985) on surgeonfish (*Acanthurus* species) and Rimmer and Wiebe (1987) on sea chub (genus *Kyphosus*) showed for the first time that marine herbivorous fish possessed distinct symbiotic gut microbiota that aided fermentative digestion. Since then, anaerobic bacterial species, frequently of the phylum Firmicutes and class Clostridia, have been repeatedly identified in the digestive tracts of herbivorous fish (Mouchet et al., 2012). Within the body of evidence, there has been some replication of species studied, with surgeonfish and sea chub repeatedly investigated (Clements et al., 1989; Clements and Choat, 1997; Mountfort et al., 2002; Moran et al., 2005). However, more recently corroborating research found that the microbiota of herbivores was distinct from that of fish with other diets and strains of Firmicutes dominated the gastrointestinal microbial communities of these fish. Published studies that supported these findings worked with a range of different species including brown-spotted spinefoot, *Siganus stellatus*, butterfish, *Odax pullus*, daisy parrotfish, *Chlorurus sordidus*, dusky parrotfish, *Scarus niger*, marblefish, *Aplodactylus arctidens*, and zebraperch, *Hermosilla azurea* (Mountfort et al., 2002; Fidopiastis et al., 2006; Clements et al., 2007; Miyake et al., 2015). Clements and associates have driven research within this field and have provided critical reviews of knowledge gained in this area thus far (Clements et al., 2009, 2014).

Gut microbial communities of fish in other trophic levels have less characteristic dominance when compared to herbivores. However, one study comparing phylogenetically similar benthivore and planktivore freshwater species showed they contained different unique intestinal bacterial communities (Uchii et al., 2006). In general, within the marine environment, Proteobacteria, rather than Firmicutes, is often the dominant phylum at the non-herbivorous trophic levels (Miyake et al., 2015). Vibrionaceae, *Aeromonas* and *Pseudomonas* are all frequently reported in carnivores, omnivores and (zoo-) planktivores. The gut microbiota of temperate pelagic planktivores such as mackerel, *Scomber scombrus*, and herring as well as tropical planktivores such as pipefish, *Syngnathus scovelli*, sardines, *Sardinella longiceps*, damselfish, *Pomacentridae*, and cardinalfish, *Apogonidae*, have all been studied (Karthiayani and Mahadeva Iyer, 1967; Hansen et al., 1992; Ransom, 2008; Svanevik and Lunestad, 2011; Parris et al., 2016). The dominant species reported were Gram-negative bacteria such as *Vibrio, Pseudomonas, Psychrobacter, Achromobacter, Shewanella, Alteromonas, Endozoicomonas*, Vibrionaceae and Pasteurellaceae (**Table 2**). Two studies that looked at omnivore species; long-jawed mudsucker, *Gillichthys mirabilis*, and pinfish, *Lagodon rhomboids*, reported *Mycoplasma* spp. as the dominant bacteria (**Table 2**; Bano et al., 2007; Ransom, 2008).

We compared 17 different published studies, which provided data on dominant gut bacteria in 16 carnivorous species and found *Vibrio* (9 times) and *Photobacterium* (7) were the most frequently reported. *Pseudomonas* was reported six different times while *Clostridium* was found in three species by five different studies. Finally, *Aeromonas, Cetobacterium, Bacillus, Mycoplasma* and *Acinetobacter* were reported twice (**Table 2**). Of these *Aeromonas, Photobacterium, Pseudomonas* and *Vibrio* have all been identified as fish gut microbiota that might aid digestion (Ray et al., 2012). *Vibrio* spp., *Enterobacter* spp., *Pseudomonas* spp. and *Aeromonas* spp. isolated from marine fish GIT have been found to produce proteases while these bacteria along with *Photobacterium* spp. have also been reported to produce chitinases (Hamid et al., 1979; MacDonald et al., 1986; Gatesoupe et al., 1997; Hoshino et al., 1997; Itoi et al., 2006). Knowledge of the principle composition of fishes gut microbiota and understanding the role they play in digestion and whole body function is critical. This is especially important as new species continue to enter the aquaculture sector and diet manipulation becomes common practise as a means to improve health and performance. The use of shotgun sequencing and transcriptome analysis in future studies will be imperative to meet this goal but it will be essential that such studies distinguish between the residential species and those which have been ingested.

### Season

Several reviews have highlighted seasonal variation and temperature changes as a defining parameter for fish gut microbial composition (Nayak, 2010; Sullam et al., 2012; Ringø et al., 2016). However, the majority of studies reporting this have been conducted on freshwater fish (Sugita et al., 1983, 1987; Macmillan and Santucci, 1990; Spanggaard et al., 2000; Al-Harbi and Uddin, 2004; Hagi et al., 2004; Naviner et al., 2006). Changes in total bacterial abundance have been reported, with peaks in summer and autumn months (Macmillan and Santucci, 1990; Al-Harbi and Uddin, 2004) as well as variations in dominant species (Hagi et al., 2004).

Historically, seasonal trends were reported in total bacterial counts recorded from gut samples from skate, *Raja* sp., and lemon sole, *Pleuronectes mimocephalus*, plated on seawater agar (Liston, 1956). This study suggested that changes in plankton availability influenced the gut bacterial composition in fish. To the best of our knowledge, the first study to directly investigate seasonal variation in gut microbiota in marine fish was by Hovda et al. (2012). The gut microbiota of adult Atlantic salmon was analysed between August and June the following year, using 16S rRNA DNA sequencing. The water temperature varied between 5.5 and 18.8°C during the experimental period. Although some bacterial species were only recorded at some of the sample time points, overall the variation reported was not statistically significant. Contrarily, a more recent study on salmon did find a relationship between seasonal water temperature changes and shifts in gut microbial composition (Neuman et al., 2016). In this study, increasing temperatures (up to 21°C) were associated with a disappearance of lactic acid bacteria (LAB) and *Acinetobacter* spp. and an increase in *Vibrio* spp. The loss of protective LAB and an increase in potentially virulent *Vibrio* spp. could have a negative impact on host health and has the potential to become an important issue with sea temperatures rising and stocks of wild salmon decreasing. Further research is required to determine the effects of seasonal variation and temperature changes on the gut microbiota of marine fish.

### Wild vs. Captured

Captive breeding and rearing of fish commonly involve the manipulation of multiple factors, including environment, social interaction and diets. Unnatural stocking densities and increased stress levels can lead to spread of disease, a major problem for the aquaculture industry (Verschuere et al., 2000). Within the sector, antibiotics have been used liberally to clear bacterial infections and even prophylactically to compensate for shortfalls in sub-standard rearing conditions (Cabello, 2006). The result of this is resistance development in aquaculture pathogens (Defoirdt et al., 2011), and reduction of microbial gut diversity in aquaculture species (Navarrete et al., 2008). Today, as regulations on the use of antibiotics in aquaculture are becoming more stringent in many countries, research into alternative methods of disease control are being prioritised. However, the aquaculture industry continues to expand and such regulations are still lax in many areas on a global scale. Assessment of the level of use and the impacts of antibiotics on aquaculture and wild fish is crucial. This is an important topic that is worthy of a full review in its own right. See the following reviews for more in depth discussion; Romero et al. (2012), Henriksson et al. (2017), and Lozano et al. (2018).

Artificial diets and increased food intake levels, often with concomitant increases in stress, can cause alterations in the microbiota in fish GIT (Clements et al., 2014). A frequently cited study that clearly depicts this relationship reports the changes in the gut microbiota of wild Atlantic cod after captive rearing (Dhanasiri et al., 2011). In this study, total counts of bacteria did not vary significantly but the diversity of bacterial species reduced notably after 6 weeks of artificial feeding. However, the study omits information on specific bacteria that are associated with the wild and subsequent captive states. Contrarily, when the gut microbiota of wild and pen-reared Atlantic salmon were compared, farmed fish had a greater microbial diversity (Holben et al., 2002). Interestingly, a novel *Mycoplasma* phylotype was found to dominate in wild Atlantic salmon and pen-reared fish in Scotland, whereas the farmed fish in Norway were dominated by *Acinetobacter junii*. The farmed fish in the two locations were fed different diets. Another study looking at changes in gut microbiota of salmon throughout the life cycle observed that all stages were dominated by Proteobacteria and were enriched for Tenericutes (Genus *Mycoplasma* especially; Llewellyn et al., 2016). Taken together, these studies suggest the presence of a core microbiota that can persist often in spite of changing factors. Other studies have also reported results to support this “core microbiota” hypothesis (Roeselers et al., 2011). In the fish model species zebrafish, it was shown that there were significant similarities in the gut microbiota found in fish collected recently from their natural habitat and those reared for generations in lab facilities. However, also observed were variations correlated to lab facility and historical connecxions between these different sites (Roeselers et al., 2011).

One of the most egregious alterations commonly encountered by farmed fish is the increasing inclusion of plant ingredients into carnivorous diets. The ability of carnivorous fish to adaptively modulate digestive functions to meet changes in diet composition is limited (Buddington et al., 1997). Feed efficiency, growth rates, whole body composition of fish and nitrogen retention were significantly, negatively affected when 80% or more fish meal was replaced by plant proteins in diets fed to juvenile turbot, *Psetta maxima* (Fournier et al., 2004). Similar results were reported in a study performed on red sea bream, *Pagrus major*, whereby the experimental diet with low fishmeal and high plant protein levels caused significant reductions in feed conversion and protein efficiency ratio, digestibility of protein and disease resistance against *Edwardsiella tarda* (Khosravi et al., 2015). Studies focussed on plant protein digestion in salmonids predominate among the published literature. Addition of plant-based proteins into salmonid diets has caused numerous intestinal disorders (Urán et al., 2008; Penn et al., 2011; Sahlmann et al., 2013). These intestinal disorders are frequently reported in conjunction with alterations in the gut microbiota (Bakke-McKellep et al., 2007; Green et al., 2013). In Atlantic salmon, soybean meal-induced enteritis was accompanied by increased numbers and diversity of gut bacteria, although numbers of LAB were reduced compared to fish on a fishmeal-based diet (Bakke-McKellep et al., 2007). Similarly, Green et al. (2013) found that salmon fed soy protein concentrate experienced intestinal disorders at high seawater temperatures and coincidently experienced increased bacterial diversity which included bacteria not normally associated with marine fish (*Escherichia* and *Propionibacterium*). Gut microbial changes related to plant-protein diets have also been recorded in other carnivorous fish species. In Atlantic cod, Gram-negative bacteria *Chryseobacterium* spp. and *Psychrobacter glacincola*, and Gram-positive bacteria belonging to *Carnobacterium*, were dominant in the GIT of fish fed soybean meal while fish fed fishmeal were dominated by Gram-positive bacteria of the genera *Brochothrix* and *Carnobacterium* only (Ringø et al., 2006). Now that a link between certain plant ingredients, changes in gut microbiota composition and intestinal disorders are recognised, concerted efforts are being made to reduce the negative impacts of these ingredients, often through supplementation and further modification of the compound diets (Barrows et al., 2008; Krogdahl et al., 2010).

Changes in gut microbiota composition attributed to captive-state have also been reported in freshwater fish (Bucio et al., 2006) as well as other marine animals (Nelson et al., 2013) and are now generally accepted. However, knowledge of the gut microbiota in wild marine fish requires more attention to provide a baseline for comparative purposes to better understand the effects of captive rearing.

## Manipulation of the Fish Gut Microbiota

The innate link between a host’s microbial community and its health status is recognised in humans and other animals and much research is now directed toward methods to manipulate these microbial communities to boost host health. Fish have not been omitted from this area of nutrition and with the growth of the aquaculture industry, there has been a growing interest in the manipulation of fish gut microbiota to improve welfare and nutrition. The principle methods of gut microbial manipulation have included the alteration of dietary proteins and lipids, as well as the addition of probiotics and prebiotics in the diet.

### Proteins

Proteins, the building blocks of the body, are involved in a plethora of chemical pathways and bodily functions. The source (Desai et al., 2012), quantity (Geurden et al., 2014) and chemical structure (Kotzamanis et al., 2007) of proteins can influence gut health and microbial composition. In Atlantic salmon dietary protein quantity has been shown to alter gut microbiota. A recent study reported an association between reduced protein levels in the diet and a more divergent microbial community structure in the gut (Zarkasi et al., 2016). Peptides and glycopeptides, released through hydrolytic digestion modulate the condition and activity of the intestinal cells as well as the residing microbiota (Świątecka et al., 2012). Altering dietary protein by providing protein hydrolysates can directly and indirectly change the hosts gut microbial community. Introduction of short peptides to the diet can directly manipulate gut microbial composition by providing suitable substrates for bacteria thus encouraging proliferation (Kotzamanis et al., 2007; Delcroix et al., 2015). Certain peptides can exhibit antimicrobial activity and thus help to protect against pathogenic bacteria (Sila et al., 2014). Indirectly, they are thought to result in rapid absorption of amino acids, decreasing splanchnic extraction, causing higher systemic amino acid levels (Manninen, 2009; Egerton et al., 2018). Single amino acids play an important role in immune defence, contributing to the synthesis of antibodies and controlling key immune regulatory pathways (Kiron, 2012). Improved immunity, often associated with dietary protein hydrolysates, can allow for the reduction in pathogenic gut microbiota (Tang et al., 2008; Bui et al., 2014; Khosravi et al., 2015). The source and the degree of hydrolysis of proteins in fish diets have been reported to alter gut microbiota in larvae. Changes in culturable bacteria, especially *Vibrio* spp., were reported with seabass, *Dicentrarchus labrax*, larvae (Kotzamanis et al., 2007). Delcroix et al. (2015) also reported significant differences in gut microbiota related to diet but did not provide details of composition.

### Lipids

Fat or oil source and composition is an area of great interest in human nutrition. A recent seminal study used a rat model to show how fat type (saturated animal lard vs. polyunsaturated fish oil) altered the gut microbiota and in turn affected white adipose tissue (WAT) inflammation (Caesar et al., 2015). Lipids are important macronutrients in the diet of fish. Investigations of dietary lipids have been long-standing. The level of lipid inclusion has been examined (Lesel et al., 1989; Ringø and Birkbeck, 1999) and Lesel et al. (1989) found that increasing lipid concentrations resulted in a more diverse gut microbial community. More importantly, for the aquaculture industry, the substitution of fish oils for different dietary plant oils has also been studied (Ringø et al., 2002; Montero et al., 2010). All natural plant oils are deficient in marine polyunsaturated fatty acids; arachidonic acid, eicosapentaenoic acid and docosahexaenoic acid (Merrifield et al., 2011). Ringø et al. (2002) found differences of the aerobic gut microbial communities of Arctic charr, *Salvelinus alpinus L*., fed soybean, linseed or marine oils. This study and others (Hardy, 1997; Lødemel et al., 2001) have shown that replacement of fish oils with plant oils can actually improve fishes’ resistance to pathogenic bacteria, for which Ringø et al. (2002) suggests the associated gut microbial change plays a role. Further research is needed on this topic to confirm the effects of lipid source, composition and concentration on fish gut microbiota. Furthermore, long-chain polyunsaturated fatty acid synthesising bacteria have been discussed in the literature for over two-decades and it is reported that they have mostly been isolated from marine sources such as seawater, fish, and sediments (Nichols and McMeekin, 2002; Yoshida et al., 2016). *Shewanella* sp. along with *Vibrio* sp. are the major PUFA-producing bacterial species isolated from the GIT of fish and invertebrates (Monroig et al., 2013). Research into potential use of such bacteria for probiotic purposes would be a novel and interesting route of investigation for fish health and nutrition.

### Probiotics

Probiotics are defined as ‘live microorganisms which, when administered in adequate amounts, provide a health benefit to the host’ (WHO and FAO, 2006). Their use in aquaculture, as an alternative to antibiotics, rose significantly as legislation was introduced that restricted the widespread use of chemicals in animal rearing (Abelli et al., 2009). Gram-positive and Gram-negative bacteria, bacteriophages, microalgae and yeasts have all been tested as potential probiotics in fish (Akhter et al., 2015). Some of the most frequently investigated probiotics include LAB *Bacillus, Lactococcus, Shewanella*, and *Aeromonas* genera (Hagi et al., 2004; Burr et al., 2005; Merrifield and Carnevali, 2014). In a recent review, Carnevali et al. (2017) listed 61 published studies that investigated the administration of probiotics to teleosts. In conjunction with the manipulation of the gut microbial composition, many studies have reported an increase in growth rates (Gatesoupe, 1991; Bagheri et al., 2008; Lobo et al., 2014) and modulation of immune status (Balcázar et al., 2006; Huang et al., 2014; Cordero et al., 2015). Thus far, trials have mostly been performed on larvae and juveniles from which positive effects in the intestinal mucosal cells and stimulation of the innate immune response have been reported (Cerezuela et al., 2011; Abid et al., 2013). However, in aquaculture the successful administration of probiotics can be difficult. Issues reported include low viability of the bacteria during processing and storage, loss from leaching in the water during feeding, as well as problems related to feed handling and preparation (Merrifield et al., 2010). Despite this, when successfully administered, probiotics have been found to reduce the cost of fish farming through improvements in fish welfare and nutrition (El-Haroun et al., 2006).

### Prebiotics

Contrarily to probiotics, prebiotics do not introduce novel microbiota into the intestinal tract, but rather are defined as ‘substrates that are selectively used by host microorganisms conferring a health benefit’ (Gibson et al., 2017). As a result, these indigestible food ingredients have been shown to enhance immune response (Torrecillas et al., 2007), improve nutrient uptake (Bongers and van den Heuvel, 2003) and increase growth and feed conversion ratios (Adel et al., 2016). There are also fewer difficulties compared to probiotics in successfully administering these supplements. The principle prebiotics used for fish are fructo-oligosaccharides, short-chain fructooligosaccharides, oligofructose, mannanoligosaccharides, trans-galactooligosaccharides, inulin, galactooligosaccharides, xylooligosaccharides, arabinoxylooligosaccharides and isomaltooligosaccharides (Ringø et al., 2016). Results of prebiotic feeding studies vary considerably (Burr et al., 2010; Zhou et al., 2010; Torrecillas et al., 2014) and it appears likely that success will be supplement and dose-dependent, with considerations for time of supplementation, culture conditions, fish species and age required (Torrecillas et al., 2014). Prebiotics are sometimes used in conjunction with probiotics, creating a nutritional mixture (synbiotic) that can provide enhanced benefits for the host (Cerezuela et al., 2011). This enhanced effect was initially hypothesised as probiotics are mainly active in the small intestine, while prebiotics influence the microbiota of the large intestine in humans (Gibson and Roberfroid, 1995). Some studies have reported supportive results to suggest an enhanced effect of synbiotics over prebiotics or probiotics alone (Rodriguez-Estrada et al., 2009; Mehrabi et al., 2012). However, there has been some disparity within the published studies (Ai et al., 2011; Geng et al., 2011).

The use of probiotics and prebiotics in aquaculture is a fast-growing area and research is building an understanding of the mechanistic pathways within which they work. For recent comprehensive reviews on this topic see Cerezuela et al. (2011), Dimitroglou et al. (2011), Torrecillas et al. (2014), Song et al. (2014), Akhter et al. (2015), Ringø et al. (2016), and Carnevali et al. (2017).

## Conclusion

Similar to mammals, the gut microbiota of fish can be recognised as an organ, in itself responsible for key physiological functions which aid health maintenance of its host. Knowledge of its composition and exact functional role in health and disease is vital given the environmental changes to which fish are being exposed, particularly in light of the growth of the aquaculture industry and rising sea temperatures as a result of climate change.

The literature on the gut microbiota of marine fish thus far has provided an understanding of many areas and we now appreciate the mechanisms of colonisation and development of the fish gut microbiota. Earlier studies had suggested that bacterial levels in the fish gut were low (Yoshimizu and Kimura, 1976), while recent studies, with the help of advanced molecular techniques including next generation sequencing technologies, have painted a different view (Zarkasi et al., 2014) and numbers have been shown to reach as high as 10^9^ cfu/g in gut content of particular species.

It has been reported that 90% of fish intestinal microbiota studied to date are composed of Proteobacteria, Bacteroidetes and Firmicutes (Ghanbari et al., 2015). However, within these phyla, studies reporting gut microbiota composition have generally conveyed conflicting results and this is undoubtedly a feature of the diversity which exists amongst fish. Such diversity in results can pose difficulties in extrapolating real and meaningful trends and correlations between gut microbial composition and the factors that shape it. Despite this, studies generated to date have enabled us to infer certain conclusions such as the dominance of particular genera where the genus *Vibrio* appears to be a key member followed closely by *Photobacterium* and *Clostridium*. However, further studies are warranted to confirm such inferences. Efforts to improve and standardise sample collection, including differentiating between allochthonous and autochthonous bacteria, and subsequent analysis should greatly benefit inter-study comparisons and add strength to the data reported. Undoubtedly next generation sequencing technologies will help this enormously, providing more comprehensive datasets.

Diet and trophic level have presented as clear influencing factors of fish gut microbial composition. It has been shown that *Clostridium* is linked to an herbivorous diet while *Vibrio* and *Photobacterium* are commonly found in carnivores. Seasonal changes and the associated changes in water temperature and captive rearing have also been shown to influence microbiota composition and certain studies have cited the detrimental impact of each. In this regard, strategies which enable the manipulation of gut microbiota composition toward that of a healthy microbiota are essential. Probiotics and prebiotics are at the forefront of this but perhaps one of the greatest impediments is the lack of baseline compositional data from healthy wild fish in their natural environments. Thus, an increased focus toward collecting such data is essential if dietary manipulation strategies are to be of full benefit. Inarguably, the need to better understand the innate relationship between gut microbiota and their fish hosts is the ultimate goal. Some excellent initial work on the role and mechanistic pathways of gut microbiota has been produced (Ringø et al., 2010; Ray et al., 2012). However, gaining a greater understanding of the specific effects of particular microbes and their associated components on host health will improve our ability to manipulate and fortify fish gut microbial communities to enhance fish health and aquaculture productivity. The use of transcriptomics will be important in this future research.

There are a number of important topics in this area that would benefit from further research in the future. Firstly, producing baseline data on the gut microbiota of wild populations, which includes domains beyond just bacteria should be prioritised. Investigations into the potential effects of on-coming climate change including changes in water salinity, acidity and temperature on the gut microbiota of fish will be important. The other area that will continue to be prioritised is diet manipulation. Finding diets that are sustainable and also benefit the fish in terms of nutrition and health is imperative for the aquaculture industry. Throughout these studies, the role of the gut microbiota will need to be considered. Finally, the supply of marine lipids is becoming an inhibitory factor for the aquaculture industry. In this review, we have highlighted two interesting areas of research related to this which are worthy of further research. Firstly, initial reports linking dietary plant oils to pathogenic resistance and secondly, the formative research on PUFA-producing bacteria that could potentially play an important role in meeting the future demands for marine lipids. Although researchers working in this field have significantly expanded our knowledge on this topic there is still great scope for further research. Data collection from wild populations, laboratory experiments and work within aquaculture will all be important contributors.

## Author Contributions

SE is responsible for the writing of this review. The other four authors SC, JW, CS, and RPR are the Ph.D. supervisors of SE and are responsible for assisting in the final drafting of the manuscript.

## Conflict of Interest Statement

The authors declare that the research was conducted in the absence of any commercial or financial relationships that could be construed as a potential conflict of interest.
